# Forensic psychiatry in the arctic – a comparative study of patient characteristics, health care system and legislation in greenland and nunavut

**DOI:** 10.1192/j.eurpsy.2021.1007

**Published:** 2021-08-13

**Authors:** C. Upfold, C. Jentz, G. Chaimowitz, P. Heilmann, N. Nathanielsen

**Affiliations:** 1 Forensic Psychiatry Program, St. Joseph’s Healthcare Hamilton, Hamilton, Canada; 2 Department Of Forensic Psychiatry, Aarhus University Hospital Psychiatry, Aarhus N, Denmark; 3 Department Of Psychiatry, Queen Ingrid’s Hospital, Nuuk, Greenland; 4 The Directorate Of Correctional Services, Probation Services in Nuuk, Nuuk, Greenland

**Keywords:** Forensic, Arctic, psychiatry, International Comparison

## Abstract

**Introduction:**

Greenland and the Canadian territory of Nunavut appear to have a different prevalence of forensic psychiatric patients, despite their comparable population and landmass sizes. Both are mainly inhabited by Inuit with a similar cultural and social background. Both have a universal health care system. They differ, however, concerning the supply of mental health services and legislation concerning forensic psychiatric patients.

**Objectives:**

To compare the prevalence and clinical characteristics of forensic psychiatric patients in Greenland and Nunavut.

**Methods:**

Data is obtained from health records, forensic psychiatric evaluations and court acts from all forensic psychiatric patients 18 years or older living in Greenland or admitted to the University Hospital Aarhus (N≈100). Data extracted from Nunavut Review Board hospital reports will be used to describe the patient population from Nunavut (N≈15). Patient characteristics include gender, age, marital status, education, diagnosis of mental illness, medical treatment, family history of mental illness and serious adverse childhood experiences. Public documents concerning health systems and legislation will be identified through literature search.

**Results:**

Patient characteristics from the two patient populations, as well as visualizations of the differences and similarities between the respective health care and legislative systems will be presented at the conference.

**Conclusions:**

This study provides a comprehensive clinical, socio-demographic and forensic comparison of the forensic psychiatric populations in Greenland and Nunavut, Canada. To our knowledge, it will be the first to describe and compare forensic psychiatric populations in the Arctic.

